# Identification of Pathogenic Variants in *CYP4F22*, *FLG*, *ALOX12B*, and *NIPAL4* in a Case Series of Inherited Ichthyosis

**DOI:** 10.3390/ijms27104639

**Published:** 2026-05-21

**Authors:** Malali Abdul Sattar, Amna Aurang Zaib, Huda Abbasi, Mirza Zain Ul Abideen, Saima Riazuddin, Zubair M. Ahmed, Muhammad Naeem

**Affiliations:** 1 Department of Biotechnology, Quaid-i-Azam University, Islamabad 45320, Pakistan; malalikakar@gmail.com (M.A.S.); abbasihuda1@gmail.com (H.A.); mirza.abideen@gmail.com (M.Z.U.A.); 2 Department of Otorhinolaryngology Head and Neck Surgery, School of Medicine, University of Maryland, Baltimore, MD 21201, USA; azaib@som.umaryland.edu (A.A.Z.); sriazuddin@som.umaryland.edu (S.R.); 3 Centre of Excellence in Molecular Biology, University of the Punjab, Lahore 54590, Pakistan; 4 Department of Biochemistry and Molecular Biology, School of Medicine, University of Maryland, Baltimore, MD 21201, USA; 5 Marlene & Stewart Greenebaum Comprehensive Cancer Center, University of Maryland, Baltimore, MD 21201, USA

**Keywords:** genodermatoses, ARCI, ichthyosis vulgaris, *CYP4F22*, *FLG*, *ALOX12B*, *NIPAL4*, consanguineous families

## Abstract

Inherited ichthyoses are clinically and genetically heterogeneous disorders of cornification caused by disruption of epidermal barrier genes involved in keratinization and lipid homeostasis. Pathogenic variants in more than 50 genes have been implicated in nonsyndromic ichthyosis vulgaris (IV) and autosomal recessive congenital ichthyosis (ARCI). Here, we investigated the genetic basis of ichthyosis in four consanguineous Pakistani families presenting with IV or ARCI phenotypes. Exome sequencing followed by segregation analysis identified pathogenic variants in four established ichthyosis-associated genes: *CYP4F22*, *FLG*, *ALOX12B*, and *NIPAL4*. Identified variants include one novel nonsense allele of *CYP4F22* (c.296G>A; p.Trp99*) and three known variants previously not reported in the Pakistani population. These known variants include a nonsense change in FLG, a frameshift allele of ALOX12B, and a missense variant in NIPAL4. Standardized phenotypic annotation using Human Phenotype Ontology terms revealed overlapping but variable clinical features across families, consistent with known genotype–phenotype heterogeneity in inherited ichthyosis. In silico protein modeling using AlphaFold2 and Ramachandran plot analysis predicted structural perturbations associated with the identified variants, supporting their pathogenic relevance. Publicly available scRNAseq datasets revealed greater heterogeneity of keratinocyte-associated expression patterns of these ichthyosis-associated genes in aging samples. Collectively, our findings expand the allelic and phenotypic spectrum of inherited ichthyosis in the Pakistani population and highlight the utility of comprehensive genetic analysis in consanguineous families for accurate molecular diagnosis, genetic counseling, and disease epidemiology.

## 1. Introduction

Ichthyoses are a clinically and genetically heterogeneous group of cornification disorders characterized by abnormal epidermal differentiation and impaired skin-barrier function, typically presenting with generalized scaling with variable erythema, inflammation, and hyperkeratosis. Inherited ichthyoses are broadly classified as syndromic, with extracutaneous manifestations, and nonsyndromic forms are limited primarily to the skin [1,2,3]. As of Jan 2026, loss-of-function pathogenic variants in 67 different genes have been associated with ichthyosis [4,5].

Among nonsyndromic inherited ichthyoses, ichthyosis vulgaris (IV) is the most common subtype and is primarily caused by loss-of-function (LoF) variants in *FLG* (OMIM: 135940). *FLG* encodes profilaggrin, a major component of keratohyalin granules that is proteolytically processed into filaggrin monomers during terminal keratinocyte differentiation. Filaggrin plays a critical role in keratin aggregation and epidermal barrier formation, as well as in the generation of natural moisturizing factors. *FLG* variants show a semidominant, dose-dependent inheritance pattern and affect approximately 1 in 250 individuals, with clinical severity ranging from mild xerosis to more extensive scaling and palmoplantar hyperlinearity [6,7,8].

In contrast, autosomal recessive congenital ichthyosis (ARCI) comprises a heterogeneous group of nonsyndromic ichthyoses that manifest at birth or early infancy with lamellar ichthyosis (LI), congenital ichthyosiform erythroderma (CIE), and related presentations, sometimes preceded by a collodion membrane. To date, gene reviews and large cohort studies have identified pathogenic variants in 14 genes associated with ARCI. The identified disease-associated variants disrupt multiple pathways required for epidermal barrier formation, including transglutamination, cornified envelope assembly, lipid transport, and epidermal lipid metabolism [3,9,10].

Within this lipid-centric ARCI network, *CYP4F22* (OMIM: 611495) encodes a cytochrome P450 enzyme with ω-hydroxylase activity that is essential for generating ω-hydroxyceramides/acylceramides. Functional studies have shown that reduced CYP4F22 enzymatic activity is linked to acylceramide deficiency and barrier failure, providing a direct biochemical bridge between genotype and ARCI pathogenesis [11]. *NIPAL4* encoding ichthyin is also implicated in epidermal lipid homeostasis, with pathogenic variants disrupting late-stage keratinocyte differentiation and barrier lipid organization [12,13]. In parallel, the ALOX12B lipoxygenase pathway is critical for epidermal barrier formation through oxidation of fatty-acid substrates at the stratum granulosum–stratum corneum interface, and both human genetics studies and animal models demonstrate that disruption of 12R-lipoxygenase (ALOX12B) compromises permeability barrier function [14,15].

Despite major advances in the genetics of ichthyosis, clinical interpretation remains challenging due to overlapping phenotypes and subtle genotype–phenotype relationships influenced by variant type, affected domain, and population background [16]. This is particularly relevant in consanguineous populations, where autosomal recessive genodermatoses are enriched. In this study, we investigated four consanguineous Pakistani families with inherited ichthyosis and identified pathogenic variants in *NIPAL4*, *ALOX12B*, *FLG*, and *CYP4F22*. By integrating segregation analysis, standardized phenotyping, and protein level annotation, this study presents a case series and identifies variants in multiple epidermal barrier genes associated with inherited ichthyosis.

## 2. Results

### 2.1. Identification of Pathogenic Variants in Four Pakistani Families with Ichthyosis

**Figure 1:**
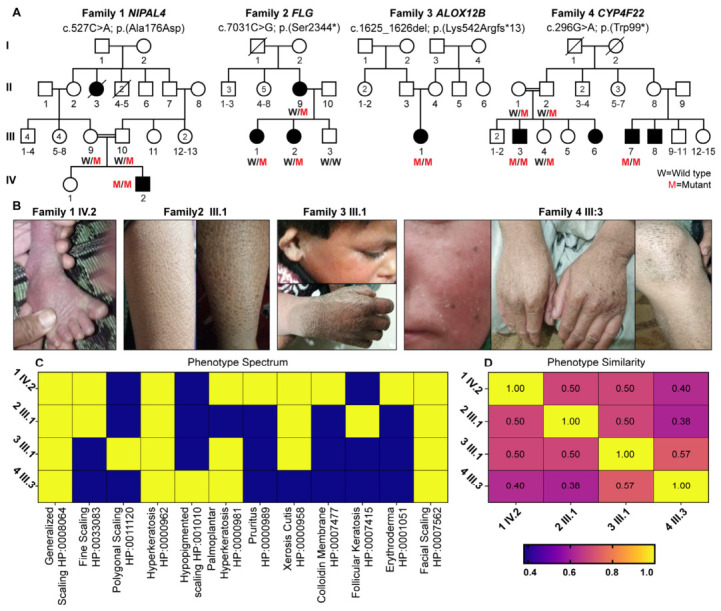
**Genetic and phenotypic spectrum in four families with inherited ichthyosis.** (**A**) Pedigrees showing segregation of pathogenic variants in *NIPAL4*, *FLG*, *ALOX12B*, and *CYP4F22* across four unrelated families. (**B**) Representative available clinical images demonstrating generalized scaling, xerosis, hyperkeratosis, and additional cutaneous features observed in affected individuals from Families 1–4. (**C**) Heatmap showing clinical features annotated using Human Phenotype Ontology (HPO) terms. Each row represents a proband, and columns represent an HPO term; yellow indicates the presence and blue indicates the absence of a feature. (**D**) Pairwise phenotypic similarity matrix calculated from HPO-based annotations. Values range from 0.38 to 1.00, with higher values indicating greater phenotypic similarity. The matrix highlights variable overlap among families despite shared ichthyosis features.

We identified four unrelated families from different geographic regions of Pakistan who were clinically diagnosed with inherited ichthyosis (Figure 1A). All affected individuals presented with abnormal cutaneous manifestations in early childhood. Exome analysis of the probands from each family identified one novel variant of *CYP4F22* (c.296G>A; p.(Trp99*)) and three known pathogenic variants (*NIPAL4*: c.527C>A; p.(Ala176Asp), *FLG*: c.7031C>G; p.(Ser2344*), and *ALOX12B*: c.1625_1626del; p.Lys542Argfs*13), which segregate with ichthyosis in the respective families (Figure 1A). Variant classification using ACMG criteria supported pathogenic or likely pathogenic status for all identified variants (Table 1 and Table S2). CADD scores ranged between 28 and 39, emphasizing that these variants are functionally damaging, which was further supported by the MutationTaster and dbNSFP scores (Table 1).

### 2.2. Phenotypic Spectrum and Similarity Across Affected Individuals

Clinical features were systematically annotated using Human Phenotype Ontology (HPO) terms, and a binary presence–absence matrix was generated for each proband and visualized as a phenotypic heatmap. Common manifestations in our ichthyosis families included generalized scaling, hyperkeratosis, erythroderma, and facial scaling, while additional features varied between individuals, reflecting phenotypic heterogeneity (Figure 1B,C; Figure S1).

Pairwise phenotypic similarity scores were calculated based on the overlap of HPO terms between affected individuals and visualized as a similarity matrix. Affected individuals from each family showed low to moderate similarity scores (0.38 to 0.57). The highest phenotypic similarity (0.57) was observed between Families 3 (*ALOX12B*) and 4 (*CYP4F22*), despite their distinct molecular etiologies. These findings highlight both shared and gene-specific phenotypic patterns within inherited ichthyosis (Figure 1D).

### 2.3. Structural Consequences of Pathogenic Variants in NIPAL4, ALOX12B, and FLG

**Figure 2:**
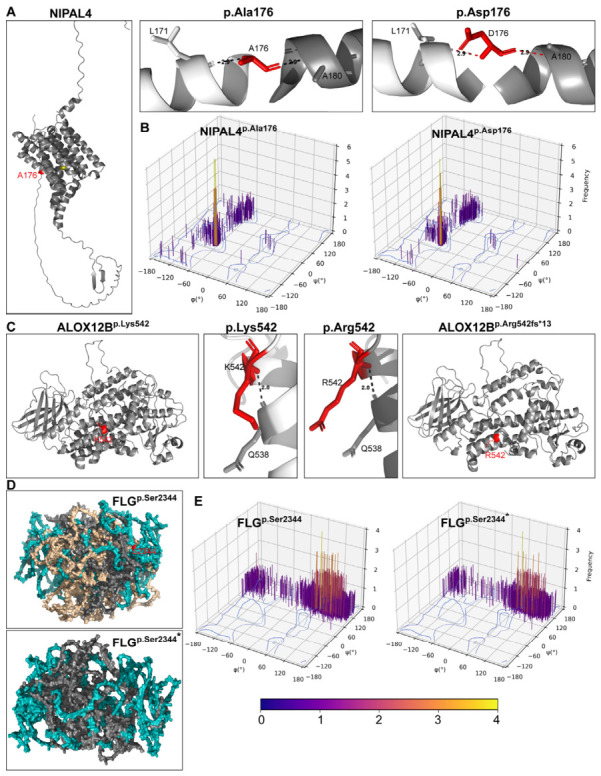
**Structural impact of pathogenic variants identified in *NIPAL4*, *ALOX12B*, and *FLG* genes.** (**A**) 3D structure modeling of NIPAL4, highlighting the p.Ala176Asp substitution. The wild-type p.Ala176 and mutant p.Asp176 residues are shown as red sticks, with neighboring residues within a 4 Å distance indicated to illustrate local structural interactions. (**B**) Ramachandran plots comparing backbone dihedral angle distributions of wild-type and mutant NIPAL4 models. (**C**) Structural models of ALOX12B showing the wild-type p.Lys542 residue and the p.Arg542 substitution (red sticks) and their interactions with surrounding residues; the p.(Lys542Argfs*13) frameshift variant is predicted to disrupt the C-terminal lipoxygenase domain. (**D**) Structural models of FLG demonstrating a compact, filamentous architecture in the wild-type protein and a disrupted, truncated structure resulting from the p.(Ser2344*) nonsense variant. (**E**) Ramachandran plots comparing wild-type and truncated FLG protein models. The Color intensity in Ramachandran plots represents residue density/frequency, with purple indicating lower density and yellow indicating higher density.

Protein structural modeling was performed to assess the potential impact of pathogenic variants on protein stability and function (Figure 2). In NIPAL4, the p.(Ala176Asp) substitution replaces a small neutral alanine with a larger, negatively charged aspartic acid within a conserved region (Figure 2A). Although interactions with adjacent residues (Leu172 and Ala180) are retained, the introduction of a negative charge is predicted to alter local electrostatic interactions and potentially affect magnesium transport. Ramachandran plot analysis indicated altered conformational distributions compared with the wild-type protein (Figure 2B). Finally, multiple sequence alignment demonstrated that p.Ala176 is highly conserved across species (Figure S2B).

Structural modeling of ALOX12B revealed that the p.Lys542Argfs*13 variant causes a frameshift that introduces a premature termination codon 13 residues downstream, producing a truncated protein. This truncation likely disrupts the C-terminal lipoxygenase domain of ALOX12B, reducing protein stability and enzymatic activity. The premature stop codon also increases the risk of nonsense-mediated mRNA decay, supporting a loss-of-function mechanism.

In FLG, p.Ser2344 is located within domain 3, which contains multiple filaggrin repeats essential for macrofibril formation and keratin aggregation. The p.Ser2344* nonsense variant introduces a premature stop codon, truncating the protein and reducing the number of filaggrin repeats from 11 to 6. Structural modeling suggests disruption of the filament-like architecture and compromised keratin bundling (Figure 2D). Ramachandran plot analysis further supported altered conformational properties in the truncated protein (Figure 2E).

### 2.4. Loss-of-Function Impact of a Novel CYP4F22 Nonsense Variant

**Figure 3:**
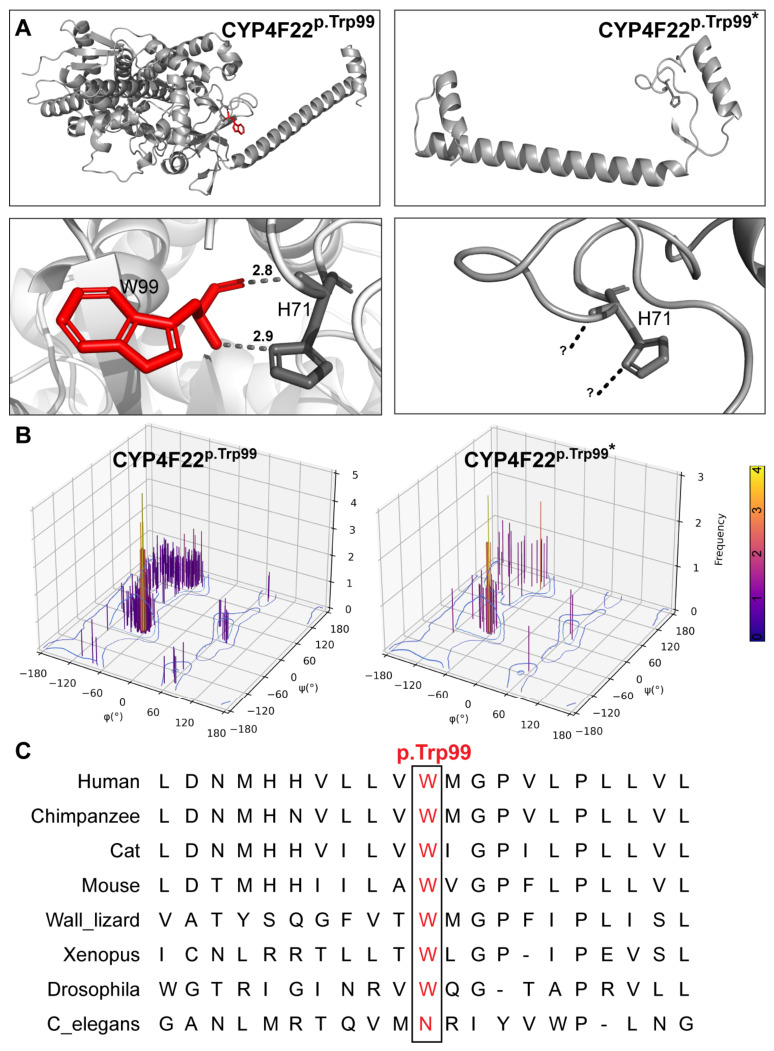
Structural and evolutionary impact of the novel CYP4F22 p.(Trp99*) variant. (**A**) AlphaFold2-predicted structures of wild-type CYP4F22 and the truncated p.(Trp99*) mutant. In the wild-type protein, p.Trp99 (red sticks) forms stabilizing interactions with p.His71, contributing to proper folding of the N-terminal region. The premature stop codon at position 99 results in loss of downstream domains and disruption of these interactions, yielding a severely truncated protein (represented as question marks (?) for lost interactions). (**B**) Ramachandran plots comparing backbone dihedral angle distributions of wild-type and p.(Trp99*) CYP4F22 models, indicating altered conformational properties in the mutant structure. (**C**) Multiple sequence alignment showing evolutionary conservation of the p.Trp99 residue across species, supporting its functional importance.

The impact of the novel *CYP4F22* (NM_173483.4) variant was evaluated by comparing wild-type and mutant protein models (Figure 3). The c.296G>A variant introduces a premature stop codon at Trp99 (p.Trp99*), likely resulting in nonsense-mediated mRNA decay and the absence of the full-length protein. In the wild-type enzyme, p.Trp99 forms stabilizing interactions with p.His71 at distances of 2.8–2.9 Å, contributing to proper folding and stability (Figure 3A). The truncated p.Trp99* model lacks most downstream domains, abolishes these stabilizing interactions, and fails to adopt a stable cytochrome P450 fold, indicating a complete loss of function (Figure 3A). Ramachandran plot analysis revealed pronounced alterations in conformational distributions compared with the wild-type protein (Figure 3B). Conservation analysis across multiple species demonstrated that p.Trp99 is highly conserved, further supporting the functional importance of this residue (Figure 3C).

### 2.5. Epidermal-Enriched Expression of Ichthyosis-Associated Genes in Human Skin

**Figure 4:**
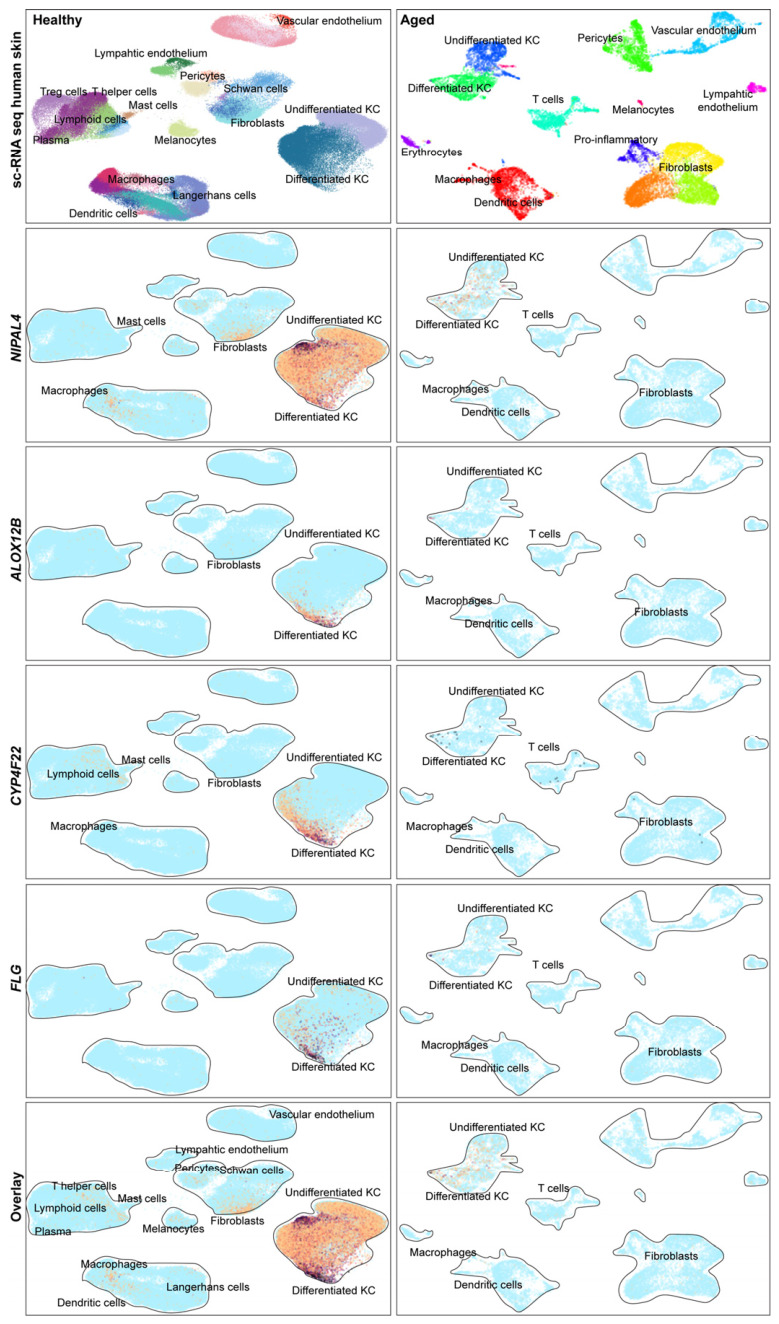
**Cellular context of ichthyosis-associated genes in healthy and aged human skin.** Single-cell RNA sequencing reference datasets from healthy adult skin and aged human skin were used to examine the cellular localization of *NIPAL4*, *ALOX12B*, *CYP4F22*, and *FLG*. UMAP projections show major skin cell populations, including keratinocytes, fibroblasts, endothelial cells, melanocytes, and immune subsets. Feature plots demonstrate that all four genes are predominantly expressed within the keratinocyte compartment, with the strongest enrichment along the differentiated keratinocyte axis. Comparison of healthy and aged skin reveals increased heterogeneity of keratinocyte-associated expression patterns in aged samples.

Publicly available single-cell RNA-seq atlases of healthy adult skin and aging human skin were examined to place ichthyosis-associated genes into a cellular context [17] The healthy skin dataset comprised transcriptomes from adult donors under homeostatic conditions (500,000 cells), while the aging skin dataset included single-cell profiles from sun-protected skin adults (~60s to 70s), encompassing ~15,000 cells [18,19] Across both datasets, *FLG*, *ALOX12B*, *CYP4F22*, and *NIPAL4* showed predominant localization within the keratinocyte compartment, with the strongest signal along the differentiated keratinocyte axis, consistent with their established roles in cornification and epidermal lipid barrier formation. Visual comparison of healthy and aged skin revealed greater heterogeneity of keratinocyte-associated expression patterns in aged samples, aligning with published evidence that aging is associated with altered skin cell-state organization and epidermal–stromal communication. This localization aligns with the known role of these genes in epidermal barrier formation and supports the pathogenic relevance of the identified variants, as disruption of keratinocyte differentiation and lipid processing is a central mechanism underlying ichthyosis phenotypes. These findings provide cell-type-specific context linking genetic variants to epidermal dysfunction (Figure 4).

## 3. Discussion

In this study, we report one novel and three pathogenic variants, previously described in other populations, of *CYP4F22*, *NIPAL4*, *ALOX12B*, and *FLG*, identified across four consanguineous Pakistani families with inherited ichthyosis, thereby expanding the geographic, allelic, and phenotypic spectrum associated with these genes. Although these genes encode proteins with distinct molecular functions, their convergence at the level of epidermal barrier integrity illustrates how disruption of different components of cornification and lipid homeostasis can give rise to clinically overlapping ichthyosis phenotypes.

The identification of a novel nonsense variant in CYP4F22 further extends the mutational landscape of ARCI-associated genes and reinforces the phenotypic variability reported for *CYP4F22*-related disease. Prior studies have demonstrated that impaired CYP4F22 enzymatic activity leads to reduced acylceramide synthesis and barrier dysfunction. However, genotype–phenotype correlations remain inconsistent, with individuals carrying identical variants exhibiting divergent clinical presentations. For *CYP4F22*, prior reports indicate that clinical presentation may vary depending on mutation type, with some patients lacking a collodion membrane at birth and developing milder phenotypes later [20,21]. Consistent with these observations, individuals in our cohort carrying a novel nonsense variant were not born with a collodion membrane and exhibited relatively milder phenotypes. Our findings are consistent with this variability and support the notion that a pathogenic variant alone may be insufficient to predict disease severity in ARCI, likely reflecting the influence of additional genetic or environmental modifiers [22,23,24,25].

Ichthyosis vulgaris can result from heterozygous, homozygous, or compound heterozygous *FLG* variants, indicating a dosage-dependent effect. In this study, the p.(Ser2344*) variant identified in the affected individual supports a dominant role for *FLG* and is consistent with previously reported dominant or semidominant inheritance patterns. The intrafamilial variability observed demonstrates incomplete penetrance and phenotypic heterogeneity in filaggrin deficiency, highlighting the challenges of clinical interpretation and the limitations of relying on clinical features to distinguish nonsyndromic ichthyosis subtypes [6,26,27,28].

Variants identified in *ALOX12B* and *NIPAL4* illustrate additional layers of heterogeneity within ARCI. Frameshift variants in *ALOX12B* are predicted to disrupt the lipoxygenase domain essential for epidermal lipid processing. *ALOX12B* prior studies suggest that loss-of-function variants are associated with more severe phenotypes, although missense variants may also show variability (Fioretti et al., 2020) [29]. In agreement with this, our patient carrying a frameshift variant (p.Lys542Argfs*13) exhibited a severe phenotype, supporting the association between truncating variants and disease severity [29]. The recurrent NIPAL4 p.(Ala176Asp) variant has been associated with variable clinical severity across different populations. In a previously reported cohort of 101 patients, the missense variant c.527C>A (p.Ala176Asp) was identified in a homozygous or compound heterozygous state in 70% of cases. Its high recurrence, together with reported ultrastructural abnormalities in lamellar body formation, supports its pathogenic relevance, while underscoring substantial phenotypic variability that cannot be explained by genotype alone. Our findings align with this variability, as affected individuals displayed features such as collodion membrane at birth, scaling, erythema, and pruritus. Notably, we also observed short stature in individuals with a missense variant, extending previous observations where this feature was associated primarily with splice-site variants [30,31,32,33,34]. Overall, these demonstrate that the phenotypes observed in our cohort are largely consistent with previously reported cases while also highlighting intra- and inter-family variability. These additions improve the integration between genetic findings and clinical presentation while maintaining appropriate caution, given the limited cohort size.

By referencing single-cell transcriptomic atlases of healthy adult and aging human skin, we show that ichthyosis-associated genes localize predominantly to differentiated keratinocyte states, reinforcing their central role in epidermal barrier formation. The increased heterogeneity observed in keratinocyte expression patterns in aged skin is consistent with age-related alterations in skin cell-state organization and suggests that barrier gene function may be modulated by broader changes in epidermal homeostasis.

Taken together, these findings underscore the complex genetic architecture of inherited ichthyosis and reinforce the value of integrated approaches combining segregation analysis, standardized phenotyping, and protein-level annotation to refine genotype–phenotype relationships. The enrichment of autosomal recessive diseases in consanguineous populations provides an important opportunity to identify rare and novel pathogenic alleles and to better delineate phenotypic boundaries associated with known genes. Although functional validation was beyond the scope of this study, future investigations using cellular or animal models will be essential to further dissect the molecular consequences of these variants and to inform the development of targeted therapeutic strategies.

## 4. Materials and Methods

### 4.1. Study Subjects and Ethical Approval

Four unrelated Pakistani families affected by inherited ichthyosis were recruited through dermatology clinics in Pakistan. The study was approved by the Institutional Review Board of Quaid-i-Azam University, Islamabad (QAU/DFBS/216). Written informed consent was obtained from all participating individuals or their legal guardians. Clinical diagnosis was based on dermatological examination and documented phenotypic features. Peripheral blood samples were collected from affected and unaffected family members. Genomic DNA was extracted using the QIAamp DNA Blood Mini Kit (Qiagen, Germantown, MD, USA) and quantified using a Quantus™ Fluorometer (Promega, Madison, WI, USA). DNA samples were normalized to a concentration of 60 ng/µL prior to sequencing.

### 4.2. Exome Sequencing and Variant Analysis

Exome sequencing (ES) was performed on one affected individual from each family (Family 1: IV-2; Family 2: III-1; Family 3: III-1; Family 4: III-3) by 3billion Inc. (Seoul, Republic of Korea). Exonic regions were captured using the xGen Exome Research Panel v2 (Integrated DNA Technologies, Coralville, IA, USA) and the Agilent SureSelect Human All Exon V6 kit (Agilent, Santa Clara, CA, USA), followed by sequencing using an Illumina NovaSeq 6000 platform (Illumina, Inc., San Diego, CA, USA). Paired-end 150 bp reads achieved a mean coverage depth of approximately 142×, covering 99% of targeted RefSeq coding regions at ≥20×. Raw data quality was assessed with FastQC, and low-quality reads or adapters were removed using Trimmomatic, discarding reads with Phred scores below 20 or shorter than 50 bp. Filtered reads were aligned to the human reference genome (GRCh37/hg19) using Burrows–Wheeler Aligner (BWA-MEM). Variant calling was performed following Genome Analysis Toolkit (GATK, version 4.6.2.0) best practices, with annotation via ANNOVAR (version 2025Mar02) and Variant Effect Predictor (VEP). Variant interpretation used EVIDENCE v4.1 (3billion Inc.), integrating pipelines for single-nucleotide variant (SNV), insertion/deletion (INDEL), and copy number variant (CNV) detection through CoNIFER v0.2.224 and 3bCNV v23.0818, enabling CNV and aneuploidy detection. We identified 67,577 SNVs and 11,247 INDELs. Variants with minor allele frequency (MAF) above 0.01 in public databases (1000 Genomes, ExAC, dbSNP, ClinVar, HGMD, and gnomAD) were excluded. Variants predicted to impact function (missense, nonsense, frameshift, splice-site) were retained. Given the consanguineous background, homozygous and compound heterozygous variants consistent with the clinical phenotype were prioritized.

Candidate variants identified by ES were validated by Sanger sequencing in the participating family members. PCR primers were designed using Primer3 software version 2.6.1 (Supplementary Table S1, Figure S2). PCR amplification and bidirectional Sanger sequencing were performed using standard protocols to confirm variant segregation within each family. Pathogenicity of identified variants was evaluated using in silico prediction tools, including MutationTaster and Combined Annotation Dependent Depletion (CADD). Evolutionary conservation was assessed using multiple sequence alignment performed with Clustal Omega (version 1.2.4). Ramachandran plots were generated using the Ramachandran Plot Server (RamPlot, 2025) to evaluate backbone φ and ψ dihedral angles of wild-type and mutant protein models. Residues were classified into favored, allowed, and disallowed regions, and differences between wild-type and mutant structures were assessed to identify conformational deviations indicative of structural perturbation.

### 4.3. Phenotypic Annotation and Similarity Analysis

Clinical features of affected individuals were annotated using standardized Human Phenotype Ontology (HPO) terms, and a binary presence–absence matrix was generated for each proband. To assess phenotypic overlap across families, pairwise phenotypic similarity scores were calculated based on shared HPO terms between probands. Similarity was expressed as a normalized score reflecting the proportion of overlapping phenotypic features relative to the total number of annotated features. The resulting similarity matrix was visualized as a heatmap to illustrate phenotypic relationships across genetically distinct cases.

### 4.4. Protein Structural Modeling and Computational Analysis

The AlphaFold database and AlphaFold 2 (ColabFold v1.6.1, default settings) were used to retrieve and predict 3D protein structures. Wild-type CYP4F22 (UniProt ID: Q6NT55), ALOX12B (UniProt ID: O75342), and NIPAL4 (UniProt ID: Q0D2K2) were obtained from the AlphaFold database. Mutant structures were predicted using AlphaFold 2 on ColabFold v1.6.1. Models with high pLDDT scores, CYP4F22 (76.2), ALOX12B (89.3), and NIPAL4 (83), were selected for visualization in PyMOL (version 3.1.8). For FLG (UniProt ID: P20930), only domain 3 was analyzed due to the protein’s large size (4061 amino acids), which exceeds AlphaFold Colab’s processing capacity. Therefore, AlphaFold-Multimer predictions were performed using the COSMIC2 cloud. Structural validation was performed using Ramachandran plot analysis (RamPlot server), where the distribution of φ (phi) and ψ (psi) backbone angles was assessed. Deviations from favored regions were used as indicators of local conformational instability in mutant proteins. PyMOL was used to assess conformational differences between wild-type and mutant models. Amino acid residues were shown as red sticks, with the protein in gray. Residues were labeled with single-letter codes, and polar contacts within 4 Å were identified. The bond lengths were compared between the wild-type and mutant residues.

### 4.5. Single-Cell RNA-Seq Data Analysis

Publicly available single-cell RNA-seq datasets from healthy adult and aging human skin were accessed via published studies and the UCSC Cell Browser. Expression of *FLG*, *NIPAL4*, *ALOX12B*, and *CYP4F22* was visualized across annotated skin cell populations to assess cellular localization without performing differential expression analyses. 

## 5. Conclusions

This study expands the geographic, allelic, and phenotypic spectrum of inherited ichthyosis by identifying pathogenic variants in *CYP4F22*, *FLG*, *ALOX12B*, and *NIPAL4* in consanguineous Pakistani families. Studying diverse populations provides new insights into ichthyosis etiology, which, in turn, can improve genetic diagnosis, genetic counseling, disease epidemiology, and personalized management.

## Figures and Tables

**Table 1:** In silico predictions and clinical details of individuals harboring Ichthyosis-associated variants.

	*NIPAL4*	*FLG*	*ALOX12B*	*CYP4F22*
Sex	Male	Female	Female	Male
Age (Years)	5	22	4	9
Ethnicities/City	Punjabi/Lahore	Punjabi/Quetta	Punjabi/Quetta	Pashtun/Quetta
Coordinate (GRCh37)	5:156895736 C>A	1:152280331 G>T	17:7978942 T>C	19:156405093 G>A
Transcript ID	NM_001099287.1	NM_002016.1	NM_001139.	NM_173483.3
cDNA change	c.527C>A	c.7031C>G	c.1625_1626del	c.296G>A
Amino acid change	p.(Ala176Asp)	p.(Ser2344*)	p.(Lys542Argfs*13)	p.(Trp99*)
Variant type	Missense	Nonsense	Frameshift del	Nonsense
Status of variant	Known	Known	Known	Novel
dbNSFP	0.634	0.215	0.241	0.288
CADD	28.6	35	38	39
MutationTaster	Disease causing	Disease causing	Disease causing	Disease causing
ACMG classification	Likely pathogenic	Pathogenic	Pathogenic	Pathogenic
ACMG evidence codes	PM2, PM1, PP1, PP3, PP4	PVS1, PM2, PP1, PP4	PVS1, PM2, PP1, PP4	PVS1, PM2, PP1, PP4
Allele frequencies (gnomAD v4.1.1)	0.0013	6.198 × 10^−7^	0.00003181	0
Total reported variants *	53	194	212	77

Abbreviations: NA: not assessed/not available, * Reported variants as of November 2025 by HGMD.

## Data Availability

The original contributions presented in this study are included in the article/Supplementary Material. Further inquiries can be directed to the corresponding authors.

## Supplementary Materials

The following supporting information can be downloaded at: https://www.mdpi.com/article/10.3390/ijms27104639/s1.
