# Supplementary material for: Identification of Pathogenic Variants in CYP4F22, FLG, ALOX12B, and NIPAL4 in a Case Series of Inherited Ichthyosis
Source: Int J Mol Sci. 2026 May 21;27(10):4639. doi: 10.3390/ijms27104639 (PMC13207361; doi:10.3390/ijms27104639)
Supplement: Supplementary file 1 [file ijms-27-04639-s001.zip › ijms-4204126-supplementary.pdf]

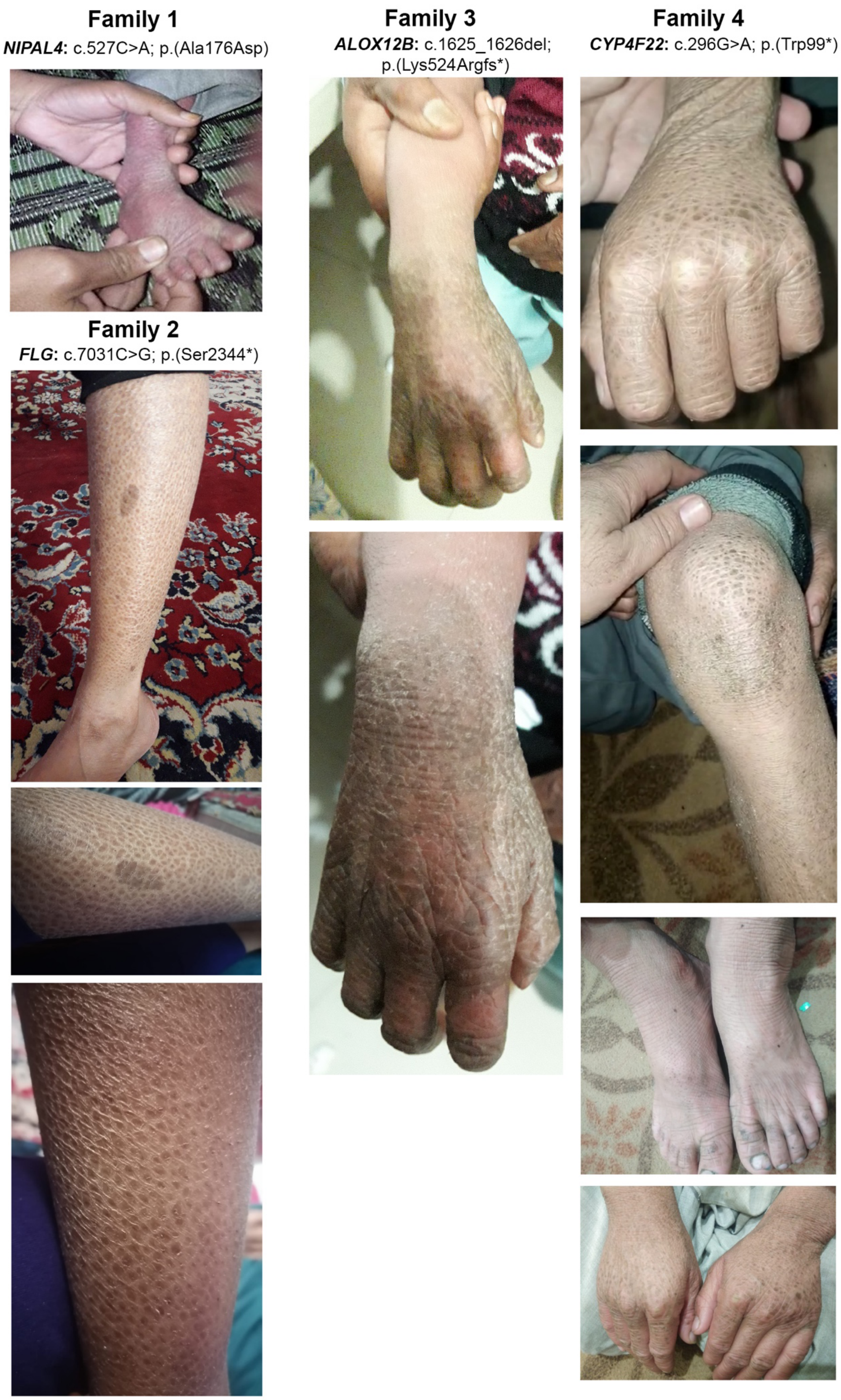

Supplementary Figure S1. Presentation of skin disorders in affected individuals.

Figure S2

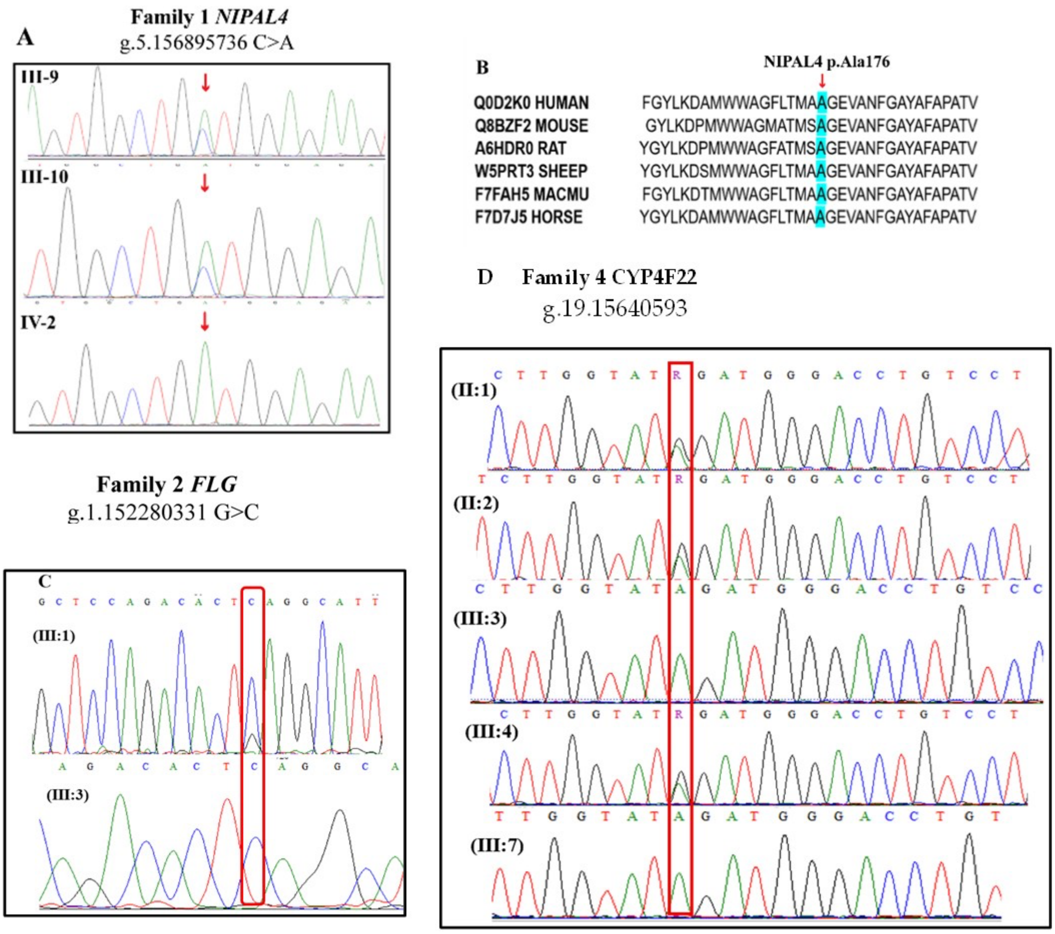

Supplementary Figure S2. Sanger validation and conservation analysis

(A) Sanger sequencing chromatograms from Family 1 (*NIPAL4*) showing the g.5:156895736 C>A variant in the patient's mother (III-9) and father (III-10), both of whom were heterozygous for the mutant allele, and the patient (IV-2) (variant position indicated by red arrows) is homozygous for the variant, This observed segregation pattern supports a recessive mode of inheritance. (B) Multiple sequence alignment of NIPAL4 protein sequences across species demonstrating conservation of the p.Ala176 residue (highlighted), supporting functional relevance of the affected site. (C) Sanger sequencing was conducted to confirm the presence of the p.(Ser2344Ter) variant in the *FLG* gene within family 2. The affected daughter (III-1) was identified as heterozygous for the variant, whereas the healthy brother (III-3) was homozygous for the wild-type allele. This observed segregation pattern supports a dominant mode of inheritance. (D) Sanger sequencing chromatograms from Family 4 (*CYP4F22*) confirming the g.19:15640593 G>A variant (red boxed); the heterozygous base call is indicated as R (Het) at the variant site.: the affected patient (III-3) and his cousin (III-7) were homozygous for the c.296G>A variant, while the unaffected mother (II-1), father (II-2), and his sister (III-4) carried the heterozygous recessive allele, This observed segregation pattern supports a recessive mode of inheritance.

Table S1: Primer sequences

Gene name	Exon	Forward primer<br>Reverse primer
NIPAL4	4	5’-TATAGCTCTGGACAGCACAC-3’<br>5’-TTCTCTGTGAGTGCTTGCTC-3’
FLG	3	5’-GACACGGATCCCACCACCAG-3’<br>5’-CTATCTACCAATTGCTCGTAGTG-3’
ALOX12B	12	5’-GAGGAGGATCCAAACTGA-3’<br>5’-GCCAGAGAAGAGCCTGTC-3’
CYP4F22	4	5’-GAGACTACAGGAGGTGGC-3’<br>5’-GAACTCTTAGTCATTGCCACATC-3’

Table S2: ACMG Justifications for Variants

**Gene**	**mutation**	**Classification**	**ACMG Criteria**	**Justification**
NIPAL4	p.Ala176Asp	Likely Pathogenic	PM2, PM1, PP1,PP3, PP4	absent in population databases, supported by deleterious computational predictions, segregation, and consistent with disease phenotype.
FLG	p.Ser2344*	Pathogenic	PVS1, PM2, PP1, PP4	Nonsense (loss-of-function) variant in a gene where LoF is a known disease mechanism, rare in population databases, segregating with a matching clinical phenotype
ALOX12B	p.Lys542Argfs*13	Pathogenic	PVS1, PM2, PP1, PP4	Frameshift variant causing predicted loss of function, Rare in population databases, segregation, and consistent clinical presentation
CYP4F22	p.Trp99*	Pathogenic	PVS1, PM2, PP1, PP4	Truncating variant leading to loss of function, absent in population databases, segregation, and consistent with ichthyosis phenotypes.
